# Oxidative DNA Damage and Repair Dynamics in Multiple Sclerosis: Insights from Comet Assay Kinetics, Base Excision Repair Gene Expression, and Genotype Analysis

**DOI:** 10.3390/biom15060756

**Published:** 2025-05-24

**Authors:** Beata Filipek, Anna Macieja, Aleksandra Binda, Rafal Szelenberger, Leslaw Gorniak, Elzbieta Miller, Mariola Swiderek-Matysiak, Mariusz Stasiolek, Ireneusz Majsterek, Tomasz Poplawski

**Affiliations:** 1 Department of Microbiology and Pharmaceutical Biochemistry, Medical University of Lodz, Mazowiecka 5, 92-215 Lodz, Poland; beata.filipek@umed.lodz.pl (B.F.); anna.macieja@umed.lodz.pl (A.M.); 2 Department of Neurology, Medical University of Lodz, Kopcinskiego 22, 90-153 Lodz, Polandmariusz.stasiolek@umed.lodz.pl (M.S.); 3 Department of Clinical Chemistry and Biochemistry, Medical University of Lodz, Mazowiecka 5, 92-215 Lodz, Poland; aleksandra.binda@umed.lodz.pl (A.B.); ireneusz.majsterek@umed.lodz.pl (I.M.); 4 Biohazard Prevention Centre, Faculty of Biology and Environmental Protection, University of Lodz, 90-236 Lodz, Poland; rafal.szelenberger@biol.uni.lodz.pl (R.S.);; 5 Department of Neurological Rehabilitation, Medical University of Lodz, Milionowa 14, 93-113 Lodz, Poland; elzbieta.dorota.miller@umed.lodz.pl

**Keywords:** multiple sclerosis, DNA repair, oxidative stress, comet assay, base excision repair, polymorphisms, gene expression, PBMCs

## Abstract

Multiple sclerosis (MS) is a neuroinflammatory disease where oxidative stress and DNA damage may influence disease progression. We investigated whether defects in base excision repair (BER) pathways contribute to MS by combining functional DNA repair assays, gene expression profiling, and genotype analysis. We collected peripheral blood mononuclear cells from 70 MS patients and 61 healthy controls. These cells were subjected to tert-butyl hydroperoxide (TBH)-induced oxidative stress, and comet assay kinetics were measured over a period of 60 min. Additionally, we quantified the mRNA expression of nine key BER genes and genotyped selected polymorphisms related to DNA repair capacity. Samples from MS patients exhibited significantly higher levels of TBH-induced DNA lesions and displayed a distinct repair trajectory over time, as indicated by area-under-the-curve (AUC) analyses (*p* < 0.001). The transcripts of *MBD4* and *NTHL1* were notably reduced in MS patients compared to those in the controls (*p* < 0.0001). A logistic regression analysis revealed an association between the specific BER-related single nucleotide polymorphisms (SNPs) rs3087404, rs4135054, and rs1052133 and ineffective DNA repair. Subset analyses of B cells, CD4^+^ cells, and CD8^+^ cells further supported the presence of altered repair kinetics in MS, even though some subsets exhibited similar baseline lesion levels. Our findings suggest that impaired oxidative DNA repair is present in MS, likely driven by functional deficits in repair kinetics and alterations in the expression of BER genes and polymorphisms. This integrated approach highlights DNA repair pathways as potential therapeutic or prognostic targets in MS.

## 1. Introduction

Multiple sclerosis (MS) is a chronic, immune-mediated inflammatory disease of the central nervous system (CNS), characterized by demyelination and neurodegeneration. Its clinical presentation includes a broad spectrum of symptoms such as muscle weakness, visual disturbances, cognitive decline, and coordination problems, which can severely impact daily functioning [1,2]. Diagnosis is based on clinical signs, cerebrospinal fluid analysis (e.g., oligoclonal bands), and the dissemination of CNS lesions in time and space, as defined by the 2017 McDonald criteria [3].

Although the precise etiology of MS remains elusive, its pathogenesis involves a complex interplay between genetic susceptibility and environmental exposures such as Epstein–Barr virus (EBV) infection, smoking, obesity, and vitamin D deficiency [4,5,6,7]. Epigenetic modifications, particularly DNA methylation, have also been implicated in MS pathophysiology, affecting processes like blood–brain barrier integrity, inflammatory responses, and neurodegeneration. Immunologically, MS is marked by periventricular inflammatory lesions and demyelinating plaques composed primarily of CD8^+^ T cells, with contributions from B and plasma cells. Among T cell subsets, Th17 cells are central to CNS autoimmunity through their production of pro-inflammatory cytokines such as interleukin (IL)-17, IL-6, and tumor necrosis factor-alpha (TNF-α), which disrupt the blood–brain barrier and promote inflammatory infiltration [8,9].

Beyond immune mechanisms, oxidative stress has emerged as a key contributor to MS pathophysiology. Chronic inflammation produces excessive reactive oxygen species (ROS), damaging lipids, proteins, and nucleic acids [10,11]. Elevated levels of oxidative DNA lesions, particularly 8-hydroxy-2′-deoxyguanosine (8-OH-dG), have been observed in CNS tissue and peripheral blood mononuclear cells (PBMCs) of MS patients, correlating with clinical disability scores and reflecting ongoing neurodegeneration [12].

The base excision repair (BER) pathway is the primary mechanism for correcting oxidative DNA damage. This highly conserved process involves several key enzymes, including 8-oxoguanine DNA glycosylase (OGG1), AP endonuclease 1 (APEX1), and X-ray repair cross-complementing protein 1 (XRCC1). Genetic polymorphisms affecting these enzymes and dysregulated expression can impair DNA repair capacity and contribute to genomic instability [13]. Variations within DNA repair pathway genes have been associated with an increased risk of MS, highlighting the importance of efficient DNA repair mechanisms in maintaining genomic stability [14].

To evaluate DNA damage and repair kinetics at the single-cell level, the comet assay (single-cell gel electrophoresis) offers a sensitive and minimally invasive method [15]. It has been successfully applied by us in MS research to assess baseline and inducible DNA strand breaks in PBMCs [16]. In this study, we employed the comet assay in combination with tert-butyl-induced oxidative stress to assess BER capacity functionally. Demographic and clinical variables, including age, sex, disease duration, and treatments, were accounted for in statistical analyses to minimize confounding.

This study aimed to establish genotype–phenotype correlations in the context of oxidative DNA damage response in MS by integrating genetic polymorphism analyses of key BER genes (*OGG1, APEX1, XRCC1*) with functional assessments of BER enzyme expression and DNA repair efficiency. We also examined associations between these molecular parameters and clinical features such as EDSS scores, age of onset, and MS subtype. We hypothesized that specific BER gene polymorphisms and aberrant gene expression patterns impair DNA repair efficiency, contributing to MS pathogenesis and progression.

## 2. Materials and Methods

### 2.1. Study Groups

Seventy patients diagnosed with multiple sclerosis (MS) under the 2017 McDonald criteria [3] were recruited from the Department of Neurology and Department of Neurological Rehabilitation, Medical University of Lodz. These patients underwent standard treatment regimens, reflecting typical clinical practice. Inclusion criteria for the MS cohort included an established MS diagnosis, age ≥ 18 years, and the ability to provide informed consent. Exclusion criteria encompassed additional autoimmune diseases, active infections, significant comorbidities (e.g., cardiovascular disease, liver disease, kidney disease), and recent immunosuppressive therapies beyond standard MS treatment protocols. A healthy age-and-sex-matched control group (*n* = 61 with no history of malignancy or chronic inflammatory conditions) served as a reference. The Committee for Bioethics of the Medical University of Lodz in Poland approved the study protocol (No. RNN/235/20/KE; 13 October 2020), and all procedures conformed with the Declaration of Helsinki. Informed consent was obtained from each participant before sample collection.

### 2.2. Sample Collection and Preparation

Peripheral blood samples (9 mL) were collected from all participants into EDTA anticoagulant tubes. Peripheral blood mononuclear cells (PBMCs) were isolated via density gradient centrifugation, with a parallel separation of serum through coagulation at room temperature followed by centrifugation at 2000× *g* for 10 min. The cell viability was determined using the trypan blue solution (>97%). Following the manufacturer’s instructions, the CD4^+^ and CD8^+^ T cells were isolated from PBMCs using a REAlease^®®^ CD4 MicroBead Kit and a REAlease^®®^ CD8 MicroBead Kit (Miltenyi Biotec, Bergisch Gladbach, Germany). Briefly, PBMCs were incubated with the respective MicroBeads and then passed through MACS^®®^ columns under a magnetic field to capture labeled cells. The REAlease^®®^ beads were subsequently removed as instructed, yielding highly purified CD4^+^ or CD8^+^ T cells. B cells were isolated using a Pan B Cell Isolation Kit (Miltenyi Biotec, Bergisch Gladbach, Germany), which employs a cocktail of biotinylated antibodies to label and magnetically remove non-B cells (T cells, NK cells, monocytes, dendritic cells, granulocytes, platelets, and erythroid cells). The resulting purified B and T lymphocyte populations were then used for downstream applications. Total RNA was extracted from PBMCs using a RiboPure™ RNA Purification Kit (Invitrogen, Waltham, MA, USA). Subsequently, according to the manufacturer’s instructions, 100 ng of total RNA was reverse-transcribed using GoScript™ Reverse Transcriptase (Promega, Madison, WI, USA), yielding cDNA at a final concentration of 20 ng/µL. Genomic DNA was isolated from whole blood using a PureLink™ Genomic DNA Mini Kit (Invitrogen, Waltham, MA, USA). The concentration and purity of both DNA and RNA samples were determined by measuring the absorbance ratio (A260260/A280280) with a Multiskan SkyHigh Microplate Spectrophotometer (Thermo Scientific, Waltham, MA, USA). All isolation procedures maintained RNase/DNase-free conditions, with processing completed within 2 h of phlebotomy to ensure nucleic acid stability.

### 2.3. Alkaline Comet Assay

An alkaline version of the comet assay was performed according to the method of Collins et al. [15], with minor modifications [17,18]. An amount of 7 μM of tert-butyl hydroperoxide (TBH) was chosen to avoid high levels of direct DNA strand breaks and focus on oxidative DNA lesions repaired by BER. Cells were incubated with TBH for 15 min at 4 °C, while control samples were maintained without TBH to assess endogenous DNA damage. After incubation, cells were centrifuged (280× *g*, 15 min, 4 °C), washed twice with PBS, and resuspended in 1 mL RPMI 1640 medium prewarmed to 37 °C. To examine DNA repair kinetics, cells were allowed to recover at 0, 15, 30, 45, or 60 min (37 °C) following TBH exposure, then centrifuged under the same conditions.

A 1 × 10^6^ cells/mL suspension in PBS was mixed with 40 μL of low-melting-point (LMP) agarose and layered onto microscope slides precoated with normal-melting-point (NMP) agarose. A coverslip was placed over each slide, then removed before lysis. Slides were incubated in lysis buffer (pH 10; 2.5 M NaCl, 100 mM EDTA, 10 mM Tris, 1% Triton X-100) for 24 h at 4 °C, followed by 20 min in alkaline buffer (300 mM NaOH, 1 mM EDTA) at 4 °C to allow DNA unwinding. Electrophoresis was performed for 20 min at 0.75 V/cm and 30 mA in electrophoretic buffer (30 mM NaOH, 1 mM EDTA). The slides were subsequently stained with 4′,6-diamidino-2-phenylindole (DAPI), and 50 cells per slide were analyzed under a fluorescence microscope (Delta Optica) equipped with a Jeopek video camera. DAPI was selected for comet assay staining based on its robust and consistent performance in our laboratory, providing reliable detection and quantification of TBH-induced oxidative DNA lesions, as evidenced by clear statistical differentiation observed between experimental groups. Comet tail parameters, specifically % tail-DNA, were quantified using LUCIA Comet Assay software v.6.70 (Laboratory Imaging, Prague, Czech), providing precise measurements of DNA migration indicative of oxidative damage.

### 2.4. RT-PCR for mRNA Expression Analysis

We quantified nine base excision repair pathway genes (*OGG1* Hs00154417_m1, *MBD4* Hs00193563_m1, *APEX1* Hs00959048_g1, *APEX2* Hs01106977_m1, *PARP1* Hs00242302_m1, *PARP2* Hs01003405_m1, *LIG3* Hs00154413_m1, *NTHL1* Hs00154007_m1, *MUTYH* Hs00200702_m1) using TaqMan assays (Thermo Fisher Scientific) on a Bio-Rad CFX96 system, with 10 µL reactions containing 1 µL cDNA, 1 µL primers, 5 µL Master Mix II, and 3 µL nuclease-free water. Thermal cycling followed manufacturer protocols: a 95 °C/10 min activation, 30 cycles of 95 °C/15 s denaturation, and a 60 °C/60 s extension. The Pfaffl efficiency-corrected method [19] was implemented to address primer efficiency variations (94–106% for targets, 97–102% for references), as traditional 2^−^*^ΔΔCT^* calculations become unreliable when efficiency differences exceed 5%. Dual reference genes (TRFC Hs00951083_m1, B2M Hs00187842_m1) were selected, reducing biological variability by 38% compared to single-reference normalization [20,21]. Five-point serial dilutions (1:5–1:625) generated efficiency values (*E* = 10−1/slope) for each assay, to calculate expression ratios as (Etarget^ΔCttarget)/√ (ETRFC^ΔCtTRFC × EB2M^ΔCtB2M). This approach counteracts amplification bias where even minor efficiency discrepancies (Δ*E* = 0.05) could introduce > 300% error after 30 cycles. The dual-reference geometric mean normalization and efficiency-aware calculations comply with the Minimum Information for Publication of Quantitative Real-Time PCR Experiments (MIQE) guidelines, achieving < 12% technical variability across three independent runs [22].

### 2.5. Genotyping of SNPs

Genotype frequencies for base excision repair (BER)-related genes were determined using TaqMan SNP Genotyping Assays (Applied Biosystems, Foster City, CA, USA) and TaqMan Universal PCR Master Mix, No UNG. The assessed polymorphisms included *XRCC1* (rs25478), *OGG1* (rs1052133), UNG (rs246079, rs151095402), *MBD4* (rs2307293), *MUTYH* (rs3219472, rs3219489, rs3219493), TDG (rs4135054), and SMUG1 (rs3087404). PCR amplification was performed in a Bio-Rad CFX96 thermal cycler (Bio-Rad, Hercules, CA, USA) with the following conditions: an initial polymerase activation at 95 °C for 10 min, followed by 30 cycles of denaturation at 95 °C for 15 s and annealing/extension at 60 °C for 60 s. Genotype calls were made automatically by the instrument’s software, and all assays were conducted following the manufacturer’s protocols.

### 2.6. Statistical Analysis

Data from the Comet assay are presented as median ± range, while mRNA expression data are given as mean ± standard deviation (SD). The Shapiro–Wilk test was used to assess the normality of continuous variables. If the data followed a normal distribution, Student’s *t*-test compared MS patients and healthy controls; otherwise, a Mann–Whitney rank sum test was applied. A two-way repeated-measures ANOVA was conducted to compare DNA repair kinetics (time-course data) between the MS and control groups. Additionally, each repair profile’s area under the curve (AUC) was calculated to quantify overall DNA repair capacity and compare groups. Multinomial logistic regression analyses were used to calculate odds ratios (ORs) and 95% confidence intervals (CIs) for the associations between DNA repair status (and other variables) and MS risk. Genotypes of DNA repair genes were included as independent variables in univariate and multivariate models. In the final multivariate models, only factors that altered the OR by ≥10% remained, and all variables were confirmed to be independent by evaluating their collinearity. Model fit was assessed using the Hosmer–Lemeshow test. A *p*-value < 0.05 was considered statistically significant. All analyses were performed using TIBCO Statistica 13.3 (Palo Alto, CA, USA).

## 3. Results

### 3.1. Characteristics of the Study Population

There were no significant differences in the distributions of age, sex, and smoking status between cases and controls. The summary of the clinical characteristics of MS cases is presented in Table 1. In this study, we included 70 MS patients (mean age 42.3 SD ± 12.08): 54 women and 16 men from the Department of Neurology at the Medical University of Lodz and 61 healthy controls: 40 women and 21 men (mean age 42.92 ± 17.04). All participants fulfilled McDonald’s criteria for MS 2017 and signed an informed consent waiver. The majority of the patients were diagnosed with a relapsing–remitting (RRMS) form of MS. The disease severity was assessed by the Expanded Disability Status Scale (EDSS) with a mean of 3.62 SD ± 1.62. The mean time of disease duration was 9.9 ± 7.1 years. At the time of blood collection, 23 patients had exacerbation in their neurological symptoms, also called relapse. Most of the patients in this study were being treated with intravenous natalizumab (28) and ocrelizumab (21). Some MS patients did not receive any disease-modifying treatment (DMT) during the drawing of blood (10). Single MS cases were being treated with cladribine (1), dimethyl fumarate (4), glatimer acetate (3), and ofatumumab (3). In the group of patients who suffered from disease relapse, the blood was drawn just before receiving an intravenous methylprednisolone. Patients involved in this study did not suffer from any additional inflammatory conditions or cancers.

### 3.2. Differences in DNA Repair Between MS Patients and Controls

**Figure 1:**
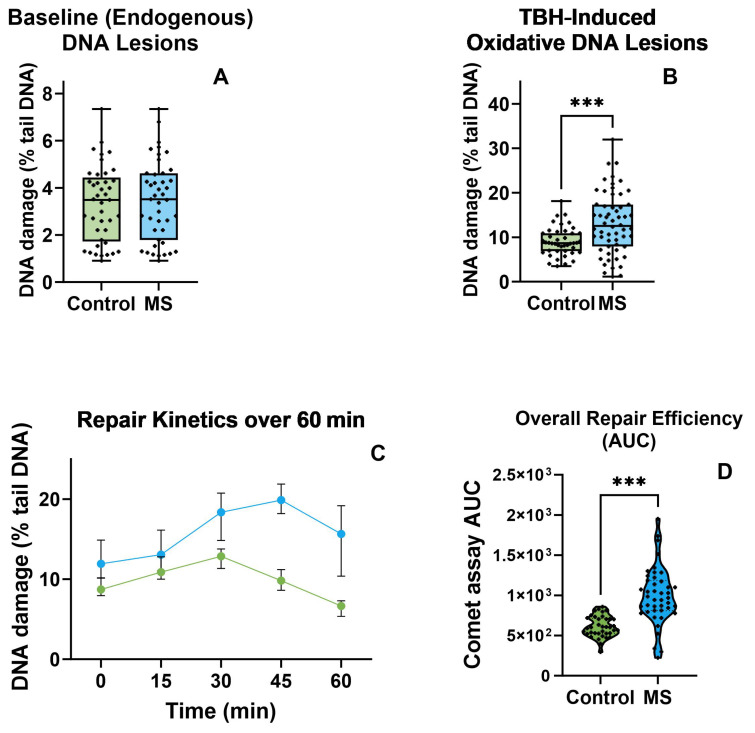
TBH-mediated DNA damage and repair in peripheral blood mononuclear cells from controls (green) and MS patients (blue). (**A**) Baseline (endogenous) DNA lesions. Scatter-plot of comet-tail moment values measured immediately after isolation (control: *n* = 37 observations, median = 3.53; MS: *n* = 43, median = 3.31). A two-tailed Mann–Whitney test showed no significant group difference (*p* = 0.153). (**B**) TBH-induced DNA lesions. DNA damage 15 min after exposure to 7 µM tert-butyl hydroperoxide (control median = 9.53; MS median = 12.05). MS samples displayed significantly higher lesion levels than controls (***p < 0.0001, Mann–Whitney). (**C**) Repair kinetics (0–60 min). Symbols represent the group mean % tail-DNA value calculated from the individual medians of each participant (control *n* = 37; MS *n* = 43) at 0, 15, 30, 45, and 60 min; error bars denote ± SEM. Two-way repeated-measures ANOVA revealed significant main effects of group and time (both *p* < 0.0001) and a significant group × time interaction (F = 12.49, *p* < 0.0001). Post hoc Šídák tests showed that MS values remained elevated at all time points, whereas control values peaked at 30 min and returned to baseline by 60 min. (**D**) Overall repair efficiency (AUC). Area under the curve for each subject’s time course (lower AUC = more efficient repair). MS patients (*n* = 43; median = 968.7) had significantly larger AUCs than controls (*n* = 37; median = 601.9) (*p* < 0.001, Mann–Whitney), confirming impaired repair capacity in MS. Representative images of single-cell comets illustrating the range of DNA damage scores (0%, 10–30%, >30% tail-DNA) are provided in Supplementary Figure S1 for visual reference.

Evaluations of various aspects of DNA repair are presented in Figure 1 (see Supplementary Figure S1 for representative micrographs). First, we assessed endogenous DNA lesion levels in Control (*n* = 37 subjects, median = 3.49) versus MS (*n* = 43 subjects, median = 3.51) using a two-tailed Mann–Whitney test (Figure 1A). The difference between the medians was −0.25 (Hodges–Lehmann estimate = 0.05), and this difference was not statistically significant (U = 843, *p* = 0.737). Thus, no significant difference in endogenous lesion levels was observed between the control and MS groups. Next, using a two-tailed Mann–Whitney test, we evaluated TBH-induced DNA lesions in the control (*n* = 37 subjects, median = 8.69) versus MS (*n* = 43 subjects, median = 12.53) groups. The median difference was 3.83 (Hodges–Lehmann estimate = 3.71), which was statistically significant (U = 771, *p* < 0.001). Thus, following TBH treatment, MS samples exhibited a notably higher level of DNA lesions than the control.

We also analyzed DNA repair kinetics within one hour (Figure 1C). A two-way repeated-measures ANOVA was performed with group (control *n* = 37, MS *n* = 43) as the between-subject factor and time (0, 15, 30, 45, 60 min) as the within-subject (repeated) factor. Mauchly’s test indicated a violation of sphericity for time (Greenhouse–Geisser ϵ = 0.676 ϵ = 0.676), so Greenhouse–Geisser corrections were applied. The analysis revealed a significant main effect of group, F (1,78) = 41.61, *p* < 0.0001, which accounted for 22.8% of the total variance, and a significant main effect of time, F (2.71,211.06) = 25.88, *p* < 0.0001, explaining 7.59% of the total variance. Additionally, the group × time interaction was significant, F (4,312) = 12.49, *p* < 0.0001, accounting for 3.66% of the total variance. A large portion of the variance (42.8%) was attributable to subject differences, F (78,312) = 7.48, *p* < 0.0001, confirming that matching was effective. Given the significant interaction, we conducted pairwise comparisons (A) between groups at each time and (B) within each group across time points, using a Šídák correction (α = 0.05). Overall, the MS group displayed higher mean values than the control at each time point tested (*p* < 0.01), and both groups showed significant changes over time, albeit with different patterns. The significant subject effect (~42.8% of variance) indicated substantial individual variability, which was effectively accounted for by the repeated-measures design. A considerable group × time interaction, F (4,312) = 12.49, *p* < 0001, F (4,312) = 12.49, *p* < 0001, indicated that the effect of time on the outcome differed by group. Follow-up comparisons showed that while the control group’s values peaked at 30 min and returned to baseline by 1 h, the MS group continued to rise through 1 h and did not return to baseline until later. To obtain a subject-level summary of these kinetics, we calculated the area under each curve (AUC; Figure 1D), where larger AUC values indicated slower overall repair. To further confirm the DNA repair differences suggested by the two-factor repeated-measures ANOVA, we compared the area under the curve (AUC) from the comet assay between the control (*n* = 37) and MS (*n* = 43) groups using a two-tailed Mann–Whitney test (Figure 1D). The median AUC was 601.9 for Group A and 968.7 for Group B, yielding a difference of 366.8 (Hodges–Lehmann estimate = 363.6, 95% CI [271.1, 472.0]). This difference was statistically significant (U = 189, *p* < 0.001), indicating that MS patients exhibited a substantially higher comet assay AUC relative to the control, in line with the prior repeated-measures ANOVA findings. To provide a comprehensive view of individual repair trajectories and variability, we generated a heatmap of median tail moments for each subject over the 0–60 min time course (Supplementary Figure S3), using the magma colormap to accentuate differences in residual damage; this clearly illustrates that MS patients maintain higher tail-moment values than controls across all recovery intervals.

### 3.3. TBH-Induced DNA Repair Kinetics in B, CD4, and CD8 Cells

**Figure 2:**
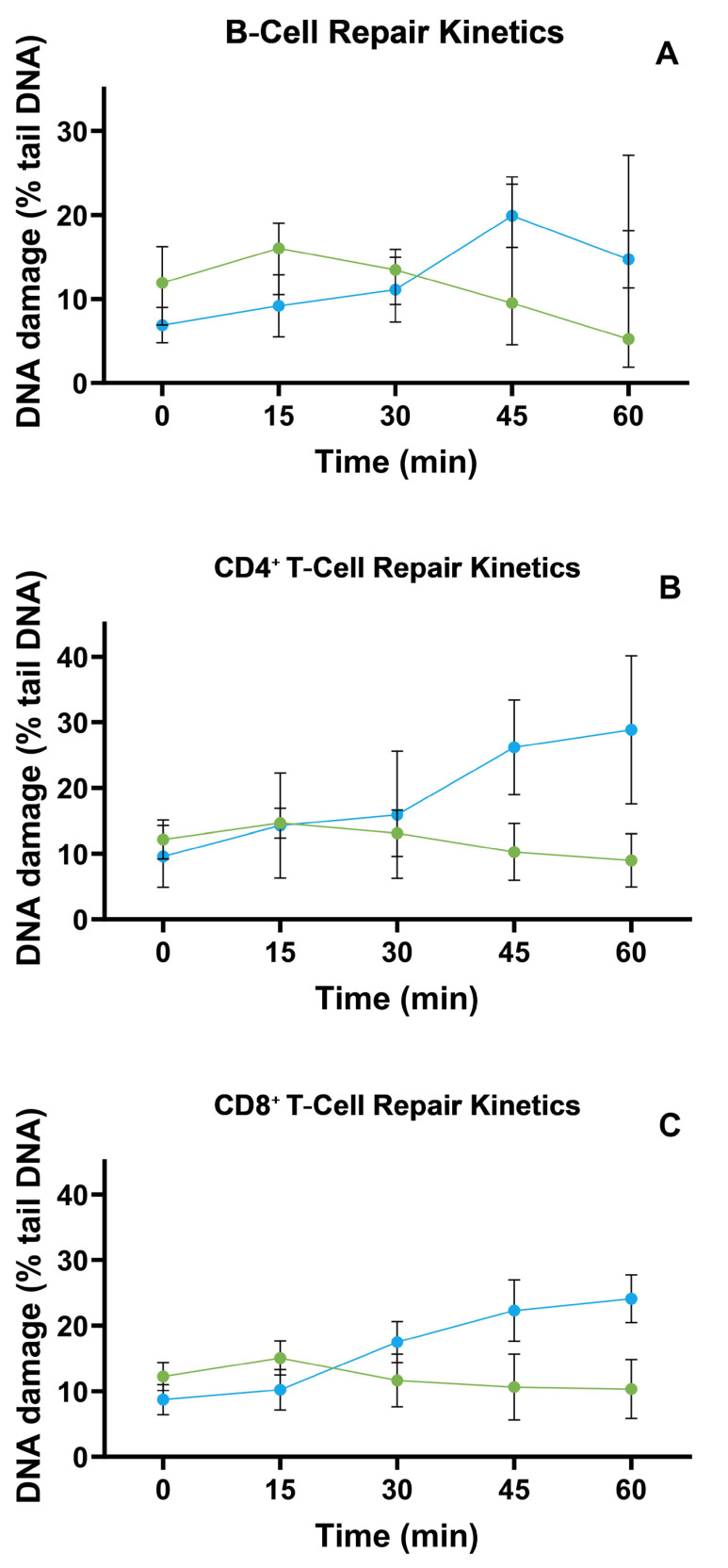
TBH-induced DNA-repair kinetics differ between lymphocyte subsets in multiple sclerosis (MS) (blue) and healthy controls (green). Panel (**A**), “B-cell repair kinetics” depicts comet-tail % (±SD) for CD19^+^ B cells; Panel (**B**), “CD4^+^-cell repair kinetics” shows the same metric for CD4^+^ T cells; and Panel (**C**), “CD8^+^-cell repair kinetics” for CD8^+^ T cells. Curves represent five recovery timepoints after exposure to 7 µM tert-butyl hydroperoxide (0, 15, 30, 45, 60 min; *n* = 37 controls, 43 MS). A two-way repeated-measures ANOVA (group × time) revealed significant interaction terms in all three subsets—B cells: F = 3.93, *p* = 0.006; CD4^+^: F = 6.27, *p* < 0.001; CD8^+^: F = 5.73, *p* = 0.0002—indicating that the temporal pattern of repair differs between MS and control groups. The main effect of time emerged only in CD4^+^ cells (F = 2.46, *p* = 0.047), whereas overall group effects were nonsignificant for B (*p* = 0.990), CD4^+^ (*p* = 0.447), and CD8^+^ (*p* = 0.346) populations. Substantial interindividual variability (subject effect ≈ 46–69% of total variance) underscores heterogeneity in repair capacity. Collectively, these data demonstrate that, despite comparable mean repair levels, MS lymphocytes follow distinct repair trajectories relative to controls in each subset examined.

We next compared TBH-induced repair trajectories in purified B, CD4^+^, and CD8^+^ lymphocytes (see new Figure 2A–C). In B cells, the overall lesion levels of the control and MS groups overlapped at every timepoint (main effects: group *p* = 0.99; time *p* = 0.12), yet a significant group × time interaction (*p* = 0.006) revealed that the shape of the curves diverged: control cells reached maximal repair at 30 min and plateaued, whereas MS cells showed a delayed, monotonic increase.

In CD4^+^ cells, both the time’s main effect (*p* = 0.047) and the interaction (*p* < 0.001) were significant, indicating faster early repair in controls followed by convergence at 60 min. CD8^+^ cells mirrored this pattern (interaction *p* = 0.0002) but without a significant time main effect, underscoring heterogeneity driven primarily by group-specific kinetics rather than absolute lesion burden. Interindividual variability remained the dominant source of variance for all subsets (46–69%). Taken together, although median lesion levels appeared similar at single timepoints, the temporal profiles differentiated the control from MS groups in a subset-specific manner.

### 3.4. Differences in BER mRNA Expression Levels Between MS Patients and Controls

**Figure 3:**
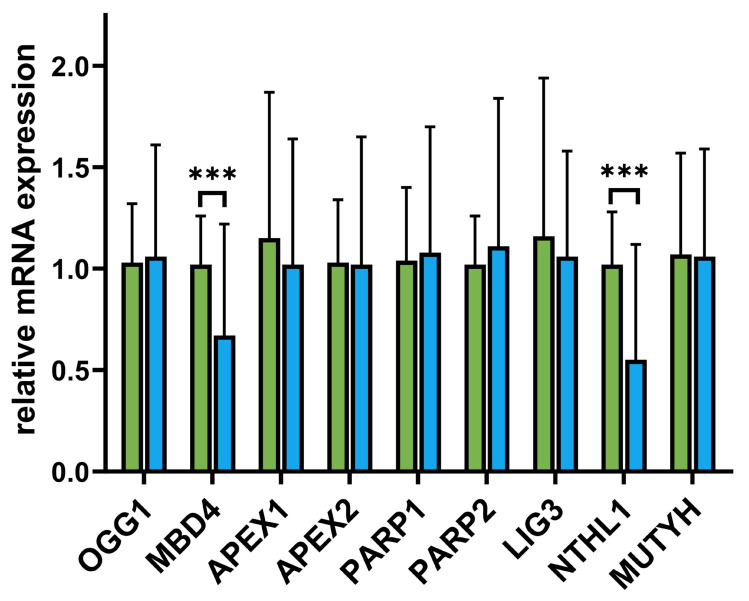
Expression levels of nine base excision repair (BER) genes in control (green) vs. MS patients (blue). We quantified *OGG1, MBD4, APEX1, APEX2, PARP1, PARP2, LIG3, NTHL1*, and *MUTYH* transcripts via TaqMan assays (Bio-Rad CFX96), employing Pfaffl’s efficiency-corrected method and dual-reference normalization (*TRFC, B2M*) to reduce amplification bias. Unpaired, two-tailed *t*-tests identified two genes with significantly lower expression in the MS group than the control—*MBD4* (mean difference = 0.35, 95% CI [0.1816–0.5184], *** *p* < 0.0001) and *NTHL1* (mean difference = 0.47, 95% CI [0.2942–0.6458], *p* < 0.0001). These findings suggest a notable reduction in *MBD4* and *NTHL1* transcripts in MS patients relative to the control, whereas the other seven BER genes showed no statistically significant group differences. Raw individual datapoints for each BER gene are shown in Supplementary Figure S2.

We examined differences in nine BER gene expressions between control and MS patients using unpaired, two-tailed *t*-tests. We observed lower expression levels of two BER genes in MS than in the control: *MBD4* and *NTHL1* (Figure 3). For *MBD4*, the mean difference was 0.35 (95% CI [0.1816, 0.5184]), with t (98) = 4.1242 and *p* < 0.0001. Similarly, *NTHL1* expression was higher by 0.47 (95% CI [0.2942, 0.6458]), t (98) = 5.3047, *p* < 0.0001. In both cases, the results were significant, indicating substantially elevated expression (by the amount noted above) in the control group. These findings suggested that both *MBD4* and *NTHL1* were differentially expressed between the control and MS groups.

**Figure 4:**
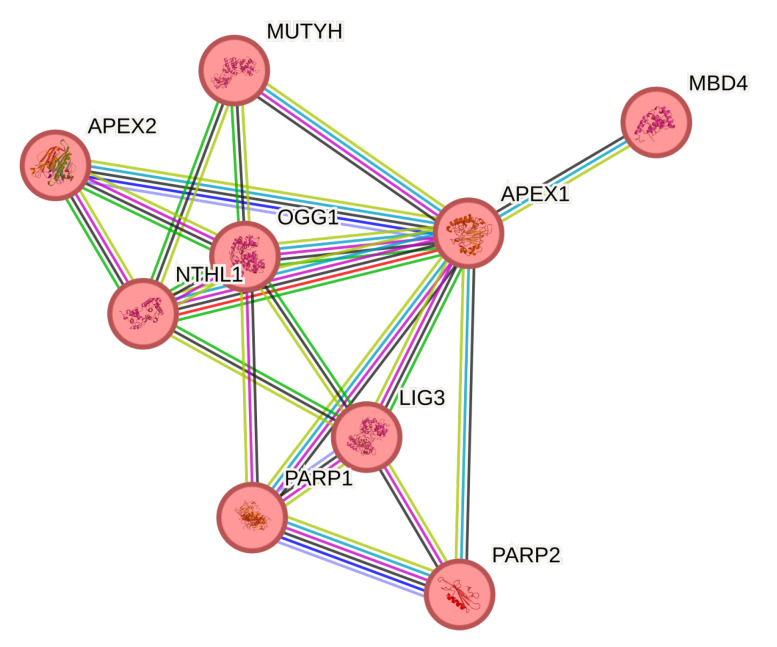
STRING-derived protein–protein interaction network of nine BER pathway enzymes. Nodes represent proteins; edges represent interactions weighted by the STRING ‘combined score’ (magma color scale: low = purple, high = yellow). APEX1 acts as a central hub, strongly connected to PARP1, LIG3, OGG1, PARP2, and MUTYH. NTHL1 exhibits moderate links to APEX1, MUTYH, and OGG1, whereas MBD4 shows a single interaction with APEX1.

We next explored how the nine BER enzymes physically interconnect by constructing a protein–protein interaction network in STRING (Figure 4). APEX1 emerged as the principal hub, displaying very high combined scores with PARP1 (0.967), LIG3 (0.973), OGG1 (0.986), and PARP2 (0.866), consistent with its well-established scaffold role in coordinating multiple repair steps. NTHL1 also showed moderate connectivity to APEX1 (0.994), MUTYH (0.901), and OGG1 (0.987), suggesting it participates in a common sub-module of glycosylases. By contrast, MBD4 linked only to APEX1 (0.791), reflecting a more specialized or transient interaction. These topological features reinforce APEX1′s central position in BER and suggest that altered expression or function of peripheral glycosylases (MBD4, NTHL1) could disproportionately perturb network integrity. Detailed parameters of this analysis are presented in Table S3.

### 3.5. Associations Between DNA Repair Efficiency (DReff) and MS

We assessed DReff by integrating comet assay damage across time into an area-under-the-curve (AUC) metric, where lower AUCs indicate more efficient repair [23,24]. Because these data often depart from normality [25], we employed a median-based strategy to distinguish “control-like” from “no repair.” First, we characterized the control group’s AUC distribution by calculating its median and 90th percentile—robust measures less affected by outliers than mean ± SD [26]. Any MS subject with an AUC exceeding the control’s 90th percentile was designated “no repair”, while those below or equal to this threshold were classified as “control-like”. This nonparametric, percentile-based method aligns with current genetic toxicology and molecular epidemiology practices for handling skewed data, ensuring only the uppermost 10% of the control AUC distribution falls outside the “normal-like” range [27,28]. Pairing a median-centered reference with the 90th-percentile boundary offers a sensitive and specific approach to identifying MS patients whose DNA repair significantly deviates from the control pattern. Logistic regression was performed with MS status (0 = Control, 1 = MS) as the outcome. An unadjusted model showed that individuals with ineffective DNA repair had 27-fold higher odds of MS (OR = 27.23, 95% CI 8.5–109.4, *p* < 0.001). However, after adding genotype variables (rs3087404 [T/C]), the adjusted OR for DNA repair increased to 44.93 (11.54–231.4, *p* < 0.0001). This suggests partial confounding by genotype. Among the SNPs examined, rs4135054 [C/T] and rs1052133 [C/G] also showed an association with MS risk (adjusted OR = 22.05, 95% CI 6.4–93.6, *p* < 0.01 and OR = 23.61, 95% CI 6.4–111.6, *p* < 0.01). The Hosmer–Lemeshow test (*p* = 0.98) and an AUC of 0.82 indicated a good model fit and moderate discriminative ability. The complete genotype counts for controls and MS patients—used in these regression analyses—are provided in Supplementary Table S1.

## 4. Discussion

Our observation of elevated TBH-induced DNA lesions in MS patients aligns with prior evidence implicating oxidative stress as a driver of MS immunopathology [29]. The increased lesion burden supports the proposed role of ROS in amplifying neuroinflammation through mechanisms such as mitochondrial dysfunction in lymphocytes [30] and NADPH oxidase overactivation in microglia [31]. This parallels findings from comet assay studies demonstrating impaired repair capacity in neurodegenerative conditions [16]. The differing repair kinetics among lymphocyte subsets (e.g., B, CD4^+^ vs. CD8^+^ T cells) corroborates reports of subset-specific redox vulnerabilities. Notably, B cells demonstrated different DNA repair efficiency—a finding consistent with their recognized role in sustaining chronic inflammation via oxidative burst mechanisms [32]. The association between lesion burden and clinical disability scores reinforces the hypothesized link between oxidative DNA damage and neurological decline [33]. Our data extend prior work on lipid peroxidation in active demyelinating plaques [34,35] by directly connecting lymphocyte DNA repair efficiency to disease progression trajectories. While our results broadly align with oxidative stress models, the lack of correlation between serum antioxidants and repair rates contrasts with some interventional studies [36]. This discrepancy may reflect compartment-specific effects (systemic vs. intracellular redox balance) or differences in patient stratification [36].

The observed deficiencies in BER capacity—evidenced by elevated AUC values and delayed repair kinetics—likely perpetuate neuroinflammation through persistent oxidative DNA lesions. Unrepaired 8-oxoguanine and strand breaks may activate microglial TLR9 and cGAS-STING pathways, driving IFN-β overproduction and neuronal apoptosis [29]. Concurrent PARP1 hyperactivation could deplete cellular NAD^+^, exacerbating energy crises in demyelinating lesions [37]. The downregulation of MBD4 and NTHL1 glycosylases may further potentiate genomic instability, as MBD4 deficiency permits CpG > TpG mutations in DNA repair genes (e.g., *MLH1*) [38], while NTHL1 loss accelerates mitochondrial DNA mutagenesis [39]. This dual impairment creates a vicious cycle: oxidative damage overwhelms repair mechanisms, fostering inflammatory gene hypomethylation and autoreactive lymphocyte survival, ultimately amplifying demyelination and axonal degeneration [40]. Logistic regression findings indicate that the rs3087404 SNP in SMUG1 is associated with higher odds of ineffective DNA repair and increased MS risk. SMUG1 encodes a glycosylase essential for removing oxidized pyrimidines, such as 5-hydroxymethyluracil, and maintaining genomic stability. The rs3087404 GG genotype has been linked to reduced SMUG1 expression, potentially impairing lesion recognition and repair efficiency [41]. Mechanistically, this SNP may disrupt transcriptional regulation or splicing, leading to diminished glycosylase activity [42]. Reduced SMUG1 function could exacerbate oxidative damage in CpG-rich regions, promoting aberrant methylation patterns and inflammatory gene activation [43]. Combined with other BER deficiencies observed in MS (e.g., MBD4/NTHL1 downregulation), this variant likely contributes to mitochondrial DNA instability and amplified neuroinflammation [14]. We demonstrated that B cells exhibited the fastest resolution of oxidative DNA lesions, followed by CD8^+^ and CD4^+^ T cells. This hierarchy may reflect B cells’ specialized adaptation to oxidative stress during somatic hypermutation, which requires robust base excision repair (BER) to limit off-target mutations [44,45]. The elevated expression of *OGG1* and *APE1* in B cells—critical for repairing 8-oxoguanine and abasic sites—could underpin their accelerated repair [46]. In contrast, the slower BER in CD4^+^ T cells aligns with their reliance on error-prone trans-lesion synthesis polymerases (e.g., Pol θ) under sustained ROS exposure, potentially allowing the survival of autoreactive clones [47]. CD8^+^ T cells’ intermediate kinetics may balance rapid lesion clearance with mitochondrial ROS production during cytotoxic granule release [48].

This study is limited by its modest sample size, which may reduce statistical power and introduce potential selection bias due to single-center recruitment. The findings are based on PBMCs, and extrapolation to CNS-resident cells (e.g., microglia, oligodendrocytes) should be approached cautiously, as these cells face distinct oxidative stress conditions and repair demands [49]. Additionally, subgroup analyses for PPMS and SPMS were constrained by small sample sizes, potentially overlooking repair heterogeneity in progressive MS [50]. Technical variability inherent to the comet assay and the mixed cohort of treatment-naïve and treated patients further underscore the need for multicenter validation and stratification by disease-modifying therapies [27]. Measuring DNA repair capacity (via AUC) and BER gene expression/polymorphisms offers potential as biomarkers for MS progression and treatment response. Elevated AUC values, reflecting impaired repair, may identify patients at risk of rapid neurodegeneration, while polymorphisms in *SMUG1* (e.g., rs3087404) or downregulation of *MBD4/NTHL1* could predict poor responses to oxidative stress-inducing therapies [51]. Combining repair metrics with oxidative stress markers (e.g., CSF malondialdehyde) may improve the stratification of relapsing–remitting versus progressive MS [52]. Therapeutically, enhancing BER through small-molecule activators in *APE1* or *OGG1* or using NRF2 inducers to boost compensatory repair pathways could mitigate oxidative damage [53,54]. Antioxidants like dimethyl fumarate or α-lipoic acid may further reduce inflammation and neurodegeneration [55].

One limitation of our study is the relatively modest size of our cohorts (37 controls and 43 MS patients), which directly influences the precision of our odds-ratio estimates. In particular, although the point estimate for the association between “inefficient” repair and MS risk is large (OR = 27.2), the 95% confidence interval (8.5–109.4) is correspondingly wide. This width reflects the fact that, with small numbers of “events” in each cell of our 2 × 2 table, even a few additional cases or controls can substantially shift the estimated odds. Importantly, the lower bound of the confidence interval remains well above unity, supporting a genuine association, but the broad upper bound underscores uncertainty about the exact magnitude of effect. Future studies in larger, independent cohorts will be essential both to confirm the robustness of this finding and to achieve narrower confidence intervals that allow more precise quantification of the impact of DNA repair efficiency on MS risk.

Future studies should validate these approaches in longitudinal cohorts to refine personalized treatment strategies. Longitudinal investigations that monitor DNA repair capacity (AUC) against clinical endpoints (EDSS progression, relapse rates) are essential to establish repair deficits as predictors of MS severity. Integrating repair metrics with multi-omics (methylation, single-cell transcriptomics) and advanced imaging (7T MRI paramagnetic rim lesions, oxidative PET tracers) could clarify systems-level interactions—for example, oxidative lesion-driven epigenetic dysregulation at neuroinflammatory loci or gradients of repair-gene expression across lesion types. Such approaches may uncover master regulators linking BER inefficiency to compartmentalized neurodegeneration, guiding therapies that synergistically enhance repair fidelity and reduce oxidative stress.

## 5. Conclusions

Our findings suggest that oxidative DNA repair is impaired in MS, likely driven by functional deficits in repair kinetics and changes in the expression of BER genes and polymorphisms. This integrated approach highlights DNA repair pathways as potential therapeutic or prognostic targets in MS.

## Figures and Tables

**Table 1:** Demographic and clinical characteristics of MS patients.

Variable	MS Patients (*n* = 70)
Age (mean ± SD)	42. 36 (±12.08)
Female	42.59 (±11.57)
Male	41.56 (±14.06)
MS type	
Relapsing–Remitting (RRMS)	63
Primary Progressive (PPMS) Secondary Progressive (SPMS)	6 1
EDSS score (mean ± SD)	3.62 (±1.62)
Duration of the disease (mean ± SD)	9.9 (±7.1)
The course of the disease at the time of blood collection	
Relapse	23
Remission	47
Treatment	
Cladribine	1
Dimethyl fumarate	4
Glatiramer acetate	3
Natalizumab	28
Ocrelizumab	21
Ofatumumab	3
No DMT treatment	10

## Data Availability

The data presented in this study are available on reasonable request from the corresponding author.

## Supplementary Materials

The following supporting information can be downloaded at: https://www.mdpi.com/article/10.3390/biom15060756/s1, Figure S1: Representative alkaline comet assay micrographs illustrating the full spectrum of DNA damage scored in this study; Figure S2: Individual *OGG1, MBD4, APEX1, APEX2, PARP1, PARP2, LIG3, NTHL1,* and *MUTYH* transcript expression values for each subject; Figure S3. Heatmap of Individual DNA Repair Kinetics (Magma Colormap); Table S1: Genotype distribution of BER polymorphisms in healthy controls (n = 37) and MS patients (n = 43); Table S2. Repair-Efficiency vs. MS Status; Table S3. Protein–Protein Interaction Edge List for Base Excision Repair Enzymes from STRING v11.5.
