# Supplementary material for: Oxidative DNA Damage and Repair Dynamics in Multiple Sclerosis: Insights from Comet Assay Kinetics, Base Excision Repair Gene Expression, and Genotype Analysis
Source: Biomolecules. 2025 May 24;15(6):756. doi: 10.3390/biom15060756 (PMC12190874; doi:10.3390/biom15060756)
Supplement: Supplementary file 1 [file biomolecules-15-00756-s001.zip › biomolecules-3617449-supplementary.pdf]

Supplementary file:

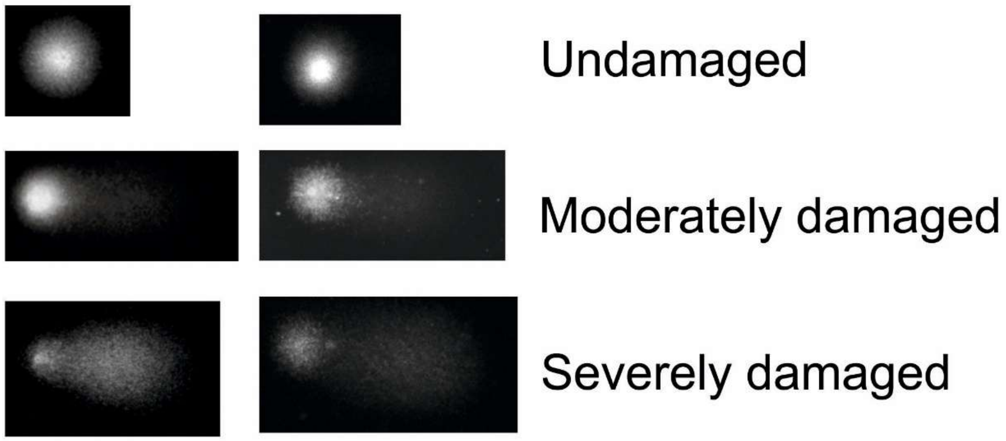

Supplementary Figure S1. Representative alkaline comet assay micrographs illustrating the full spectrum of DNA damage scored in this study.

- Undamaged PBMC (0–1 % tail DNA) collected from a healthy control before any treatment; the nucleus remains spherical with virtually no comet tail.
- Moderately damaged PBMC (~10–30 % tail DNA) observed in a multiple-sclerosis (MS) sample immediately after exposure to 7 µM tert-butyl hydroperoxide (TBH); a discernible but compact tail is visible.
- Severely damaged PBMC (> 30 % tail DNA); extensive DNA migration forms a pronounced tail.

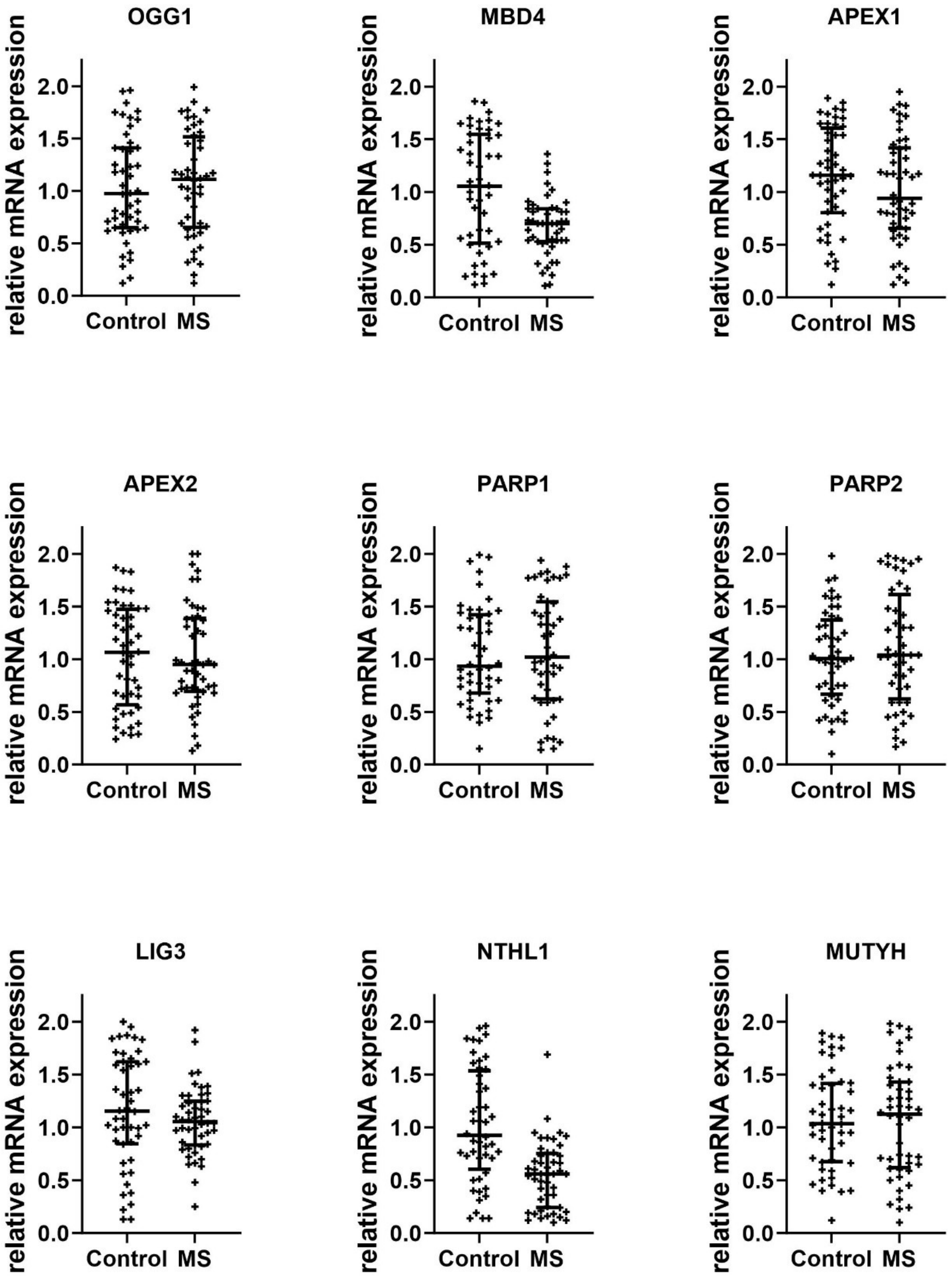

Supplementary Figure S2. Individual OGG1, MBD4, APEX1, APEX2, PARP1, PARP2, LIG3, NTHL1, and MUTYH transcript expression values for each subject. Dots represent Pfaffl efficiency-corrected expression ratios (E^ΔCt) for controls (n = 47) and MS patients (n = 53). Horizontal line = group median; vertical line = IQR

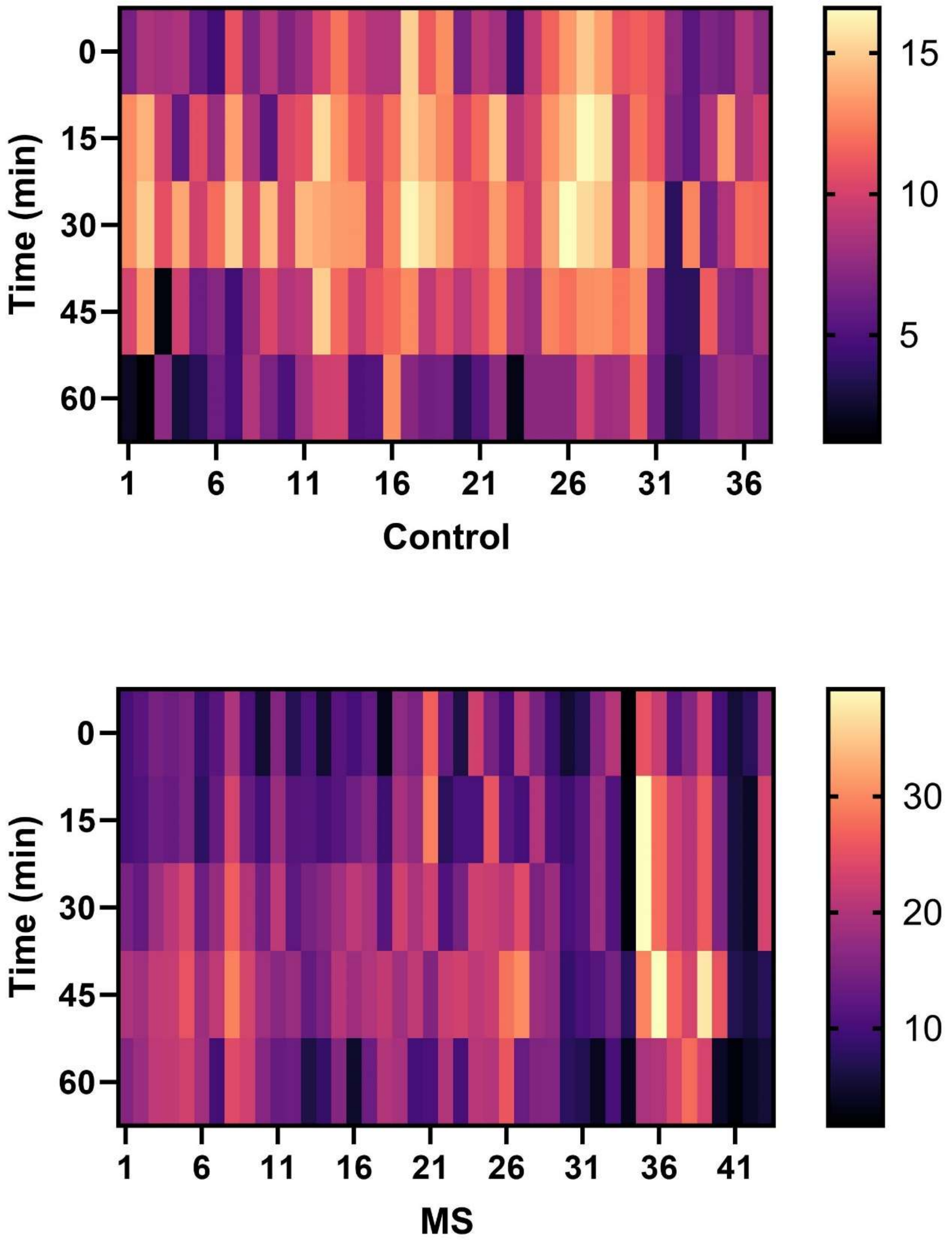

Supplementary Figure S3. Heatmap of Individual DNA Repair Kinetics (Magma Colormap). Median % DNA in tail values for each subject (columns; 37 Controls, 43 MS patients) are plotted across recovery times (rows: 0, 15, 30, 45, 60 min) following TBH treatment. The magma color scale runs from black to dark purple (low residual damage) to bright orange-yellow (high residual damage), highlighting both inter-individual variability and the sustained elevation of DNA damage in the MS group over the 60-min repair period.

Supplementary Table S1. Genotype distribution of BER polymorphisms in healthy controls (n = 37) and MS patients (n = 43).

SNP	ID	Genotype	Control_n	Control_%	MS_n	MS_%
rs25478	XRCC1	G/G	31	83.78	41	95.35
		G/T	5	13.51	2	4.65
		T/T	1	2.70	0	0.00
rs1052133	OGG1	C/G	34	91.89	18	41.86
		C/C	2	5.41	23	53.49
		G/G	1	2.70	2	4.65
rs246079	UNG	A/G	19	51.35	18	41.86
		A/A	12	32.43	14	32.56
		G/G	6	16.22	11	25.58
rs151095402	UNG	C/C	31	83.78	41	95.35
		C/T	4	10.81	2	4.65
		T/T	2	5.41	0	0.00
rs2307293	MBD4	C/C	37	100.00	41	95.35
		C/T	0	0.00	1	2.33
		T/T	0	0.00	1	2.33
rs3219472	MUTYH	C/C	24	64.86	32	74.42
		C/T	12	32.43	10	23.26
		T/T	1	2.70	1	2.33
rs3219489	MUTYH	C/C	23	62.16	32	74.42
		C/G	13	35.14	10	23.26
		G/G	1	2.70	1	2.33
rs3219493	MUTYH	C/C	32	86.49	32	74.42
		C/G	5	13.51	10	23.26
		G/G	0	0.00	1	2.33
rs4135054	TDG	C/C	28	75.68	28	65.12
		T/T	8	21.62	2	4.65
		C/T	1	2.70	13	30.23
rs3087404	SMUG1	T/T	30	81.08	21	48.84
		T/C	1	2.70	17	39.53
		C/C	6	16.22	5	11.63

Supplementary Table S2. Repair-Efficiency vs. MS Status

Repair Efficiency	Controls (n=37)	MS Patients (n=43)
Efficient (0)	32 (86.5 %)	15 (34.9 %)
Inefficient (1)	5 (13.5 %)	28 (65.1 %)

Supplementary Table S3. Protein–Protein Interaction Edge List for Base Excision Repair Enzymes from STRING v11.5.

This table lists all pairwise interactions among the nine BER pathway proteins (APEX1, APEX2, PARP1, PARP2, LIG3, OGG1, MUTYH, NTHL1, MBD4) retrieved from the STRING database. Columns include:

1. #node1

2. node2
3. node1_string_id
4. node2_string_id
5. neighborhood_on_chromosome
6. gene_fusion
7. Phylogenetic cooccurrence
8. Homology
9. Coexpression
10. experimentally_determined_interaction
11. database_annotated
12. automated_textmining
13. combined_score

1	2	3	4	5	6	7	8	9	10	11	12	13
APEX1	MBD4	9606.ENSP00000216714	9606.ENSP00000249910	0	0	0	0	0.060	0	0.500	0.591	0.791
APEX1	PARP2	9606.ENSP00000216714	9606.ENSP00000250416	0	0	0	0	0.139	0	0.500	0.715	0.866
APEX1	APEX2	9606.ENSP00000216714	9606.ENSP00000364126	0	0	0.188	0.649	0.056	0	0.900	0.333	0.942
APEX1	PARP1	9606.ENSP00000216714	9606.ENSP00000355759	0	0	0	0	0.266	0.115	0.500	0.912	0.967
APEX1	LIG3	9606.ENSP00000216714	9606.ENSP00000367787	0.087	0	0	0	0.126	0.095	0	0.968	0.973
APEX1	MUTYH	9606.ENSP00000216714	9606.ENSP00000500891	0	0	0	0	0.061	0.647	0.500	0.870	0.975
APEX1	OGG1	9606.ENSP00000216714	9606.ENSP00000306561	0.115	0	0	0	0.056	0.134	0.500	0.969	0.986
APEX1	NTHL1	9606.ENSP00000216714	9606.ENSP00000219066	0.100	0.897	0	0	0.139	0.314	0.500	0.846	0.994
APEX2	NTHL1	9606.ENSP00000364126	9606.ENSP00000219066	0.100	0	0	0	0.127	0.314	0	0.587	0.747
APEX2	OGG1	9606.ENSP00000364126	9606.ENSP00000306561	0.115	0	0	0	0.152	0.134	0	0.665	0.753
LIG3	NTHL1	9606.ENSP00000367787	9606.ENSP00000219066	0.106	0	0	0	0.049	0	0	0.702	0.724
LIG3	PARP2	9606.ENSP00000367787	9606.ENSP00000250416	0	0	0	0	0.072	0.465	0	0.984	0.991
LIG3	OGG1	9606.ENSP00000367787	9606.ENSP00000306561	0.043	0	0	0	0.253	0	0	0.875	0.903
LIG3	PARP1	9606.ENSP00000367787	9606.ENSP00000355759	0	0	0	0.548	0.107	0.641	0	0.973	0.990
MUTYH	NTHL1	9606.ENSP00000500891	9606.ENSP00000219066	0.202	0	0	0	0.181	0	0	0.861	0.901
MUTYH	OGG1	9606.ENSP00000500891	9606.ENSP00000306561	0.049	0	0	0	0.147	0	0	0.970	0.974
NTHL1	OGG1	9606.ENSP00000219066	9606.ENSP00000306561	0.061	0	0	0	0.060	0.143	0	0.985	0.987
OGG1	PARP1	9606.ENSP00000306561	9606.ENSP00000355759	0	0	0	0	0.060	0.095	0	0.794	0.809
PARP1	PARP2	9606.ENSP00000355759	9606.ENSP00000250416	0	0	0.098	0.870	0.130	0.613	0.800	0.513	0.965

- node1, node2: Gene symbols for interacting proteins
- node1_string_id, node2_string_id: Ensembl protein IDs
- neighborhood_on_chromosome, gene_fusion, phylogenetic_cooccurrence, homology, coexpression, experimentally_determined_interaction, database_annotated,

automated_textmining: Individual evidence channels (0 = no evidence; value ∈ [0–1] indicates strength)

- combined_score: Overall confidence score integrating all evidence (0–1) used for network visualization in Figure 4.
